# Genotype–Phenotype Correlations in Alport Syndrome—A Single-Center Experience

**DOI:** 10.3390/genes15050593

**Published:** 2024-05-07

**Authors:** Ștefan Nicolaie Lujinschi, Bogdan Marian Sorohan, Bogdan Obrișcă, Alexandra Vrabie, Gabriela Lupușoru, Camelia Achim, Andreea Gabriella Andronesi, Andreea Covic, Gener Ismail

**Affiliations:** 1 Department of Nephrology, “Carol Davila” University of Medicine and Pharmacy, 020021 Bucharest, Romania; bogdan.sorohan@umfcd.ro (B.M.S.); bogdan.obrisca@umfcd.ro (B.O.); alexandra.vornicu@drd.umfcd.ro (A.V.); gabriela.lupusoru@umfcd.ro (G.L.); camelia.achim@umfcd.ro (C.A.); andreea.andronesi@umfcd.ro (A.G.A.); gener.ismail@umfcd.ro (G.I.); 2 Department of Nephrology, Fundeni Clinical Institute, 022328 Bucharest, Romania; 3 Faculty of Medicine, “Grigore T. Popa” University of Medicine and Pharmacy, 700115 Iasi, Romania; andreea.covic@gmail.com; 4 Nephrology Depatment, Dialysis and Renal Transplant Center, “Dr. C. I. Parhon” Clinical Hospital, 700503 Iasi, Romania

**Keywords:** monogenic kidney diseases, Alport syndrome, type IV collagen-related disorders, genotype–phenotype correlations, kidney survival

## Abstract

Background: Alport syndrome (AS) is a common and heterogeneous genetic kidney disease, that often leads to end-stage kidney disease (ESKD). Methods: This is a single-center, retrospective study that included 36 adults with type IV collagen (COL4) mutations. Our main scope was to describe how genetic features influence renal survival. Results: A total of 24 different mutations were identified, of which eight had not been previously described. Mutations affecting each of the type IV collagen α chains were equally prevalent (33.3%). Most of the patients had pathogenic variants (61.1%). Most patients had a family history of kidney disease (71%). The most prevalent clinical picture was nephritic syndrome (64%). One-third of the subjects had extrarenal manifestations, 41.6% of patients had ESKD at referral, and another 8.3% developed ESKD during follow-up. The median renal survival was 42 years (95% CI, 29.98–54.01). The COL4A4 group displayed better renal survival than the COL4A3 group (*p* = 0.027). Patients with missense variants had higher renal survival (*p* = 0.023). Hearing loss was associated with lower renal survival (*p* < 0.001). Conclusions: Patients with COL4A4 variants and those with missense mutations had significantly better renal survival, whereas those with COL4A3 variants and those with hearing loss had worse prognoses.

## 1. Introduction

Alport syndrome (AS) is a common and heterogeneous genetic kidney disease, characterized by structural defects of the glomerular, cochlear, and ocular basement membranes leading to renal dysfunction, sensorineural hearing loss, and ocular abnormalities [1]. Although traditionally considered a rare disease, with a highly geographically variable prevalence [2,3], recent studies suggest that nearly 1% of the population could be at risk of having a heterozygous pathogenic variant of the genes coding the α 3 and α 4 chains of type IV collagen molecules (COL4A3 and COL4A4) [4].

The natural evolution of kidney involvement in AS is usually divided into a series of stages: microscopic hematuria, proteinuria, and progressive chronic kidney disease (CKD) [5,6,7]. Males with X-linked AS (XLAS) and patients with autosomal recessive AS (ARAS) display a worse clinical picture, with early onset end-stage kidney disease (ESKD) associated with extrarenal manifestations, whereas autosomal dominant forms of AS (ADAS) and females with XLAS usually exhibit a milder phenotype [5,6,7].

In recent years, the acknowledgement of a higher prevalence of ADAS has led to a paradigm shift regarding the clinical spectrum of type IV collagen disorders. It is now recognized that some forms of AS can present as focal and segmental glomerulosclerosis (FSGS) rather than the typical clinical picture [8,9,10]. Due to this large phenotypic variability, making a correct diagnosis based only on clinical features is often difficult [7], as shown by Groopman et al., who observed that only 38% of patients with variants involving type IV collagen genes (COL4) in their cohort had a correct clinical diagnosis prior to genetic testing [11]. In the same study, 16% of patients with AS had previously been diagnosed with FSGS based on clinical and pathological criteria [11].

As genetic testing becomes more readily available, there is a growing interest in the correlations between the molecular characteristics of each variant and clinical outcomes. Previous studies have already demonstrated that patients with truncating variants and large deletions have a worse prognosis, with kidney replacement therapy (KRT) initiated at younger ages [12,13,14]. For missense variants, which tend to have a more heterogeneous phenotype, there are multiple factors that can influence renal outcomes: the position of the substitution, the nature of the substituted amino acid, and the molecular characteristics of the substituting residue [15,16]. Therefore, apart from inheritance, the molecular impact of each variant can be a valuable tool for individualized prognostication.

The present study aims to describe how these genetic features influence renal survival in patients with AS followed in a tertiary center.

## 2. Materials and Methods

### 2.1. Study Design and Objectives

Forty-nine patients with COL4 variants were identified in our database between January 2020 and April 2023. For 36 of them, complete medical histories and laboratory data were available. We conducted a retrospective analysis aiming to describe the clinical spectrum of COL4-related disorders and how genetic features influence renal survival. The inclusion criteria were as follows: (1) subject age > 18 years and (2) genetic diagnosis of a COL4-related disorder (i.e., pathogenic [PV], or likely pathogenic variants [LPV] involving the COL4 genes, regardless of the clinical phenotype; variants of uncertain significance [VUS] only in the presence of clinical and/or histological features suggestive of AS or FSGS.

### 2.2. Clinical Data

Clinical data were retrospectively collected from medical records. Clinical variables included sex, age at the time of clinical onset, age at the time of genetic testing, clinical picture at onset, family history of kidney disease if present, and treatment history focusing on the use of immunosuppressive therapies, renin-angiotensin-aldosterone system inhibitors (RAASis) and sodium-glucose cotransporter 2 inhibitors (SGLT2is). Laboratory data included serum creatinine, estimated glomerular filtration rate (eGFR), and urinalysis results. The main events recorded were the occurrence of ESKD and the initiation of KRT. For subjects who underwent a kidney biopsy, depending on the findings from light microscopy (LM) and electron microscopy (EM), the results were classified into five categories: suggestive of (1) AS, (2) thin basement membrane disease (TBM), (3) FSGS, (4) vascular lesions and (5) other nephropathies. If EM was available, the presence of podocyte foot process effacement and glomerular basement membrane (GBM) alteration (i.e., thinning, thickening, and splitting of the GBM with a multi-laminated appearance) were recorded. To assess the renal function at the time of referral, the 2021 CKD Epidemiology Collaboration (CKD-EPI) creatinine equation [17] was used. ESKD was defined as eGFR under 10 mL/min/1.73 m^2^ or the need for KRT initiation.

### 2.3. Genetic Testing

A complete description of the genetic variants was recorded for each patient. Genetic testing was provided by two accredited laboratories. Pathogenicity was assessed by the laboratory, or if unspecified, by using VarSome tools [18]. Variant classification was performed according to the guidelines of the American College of Medical Genetics and Genomics (ACMG) [19]. Patients with variants classified as VUS were included only if the clinical and/or histological features were highly suggestive of AS or FSGS. Zygosity and inheritance were assessed by the laboratory. When more than one distinct α chain was affected, patients were labeled as having a digenic or a complex inheritance pattern. Based on previous studies regarding the types of mutations responsible for XLAS, variants were classified into the following categories: (1) missense, (2) nonsense, (3) frameshift, (4) in frame deletions and insertions, (5) noncoding, and (6) splice junction loss [20,21]. Missense variants were stratified into (1) glycine substitutions or (2) substitutions involving another residue. In the case of glycine substitutions, depending on the degree of instability caused by the substituting residue, variants were classified as (1) mildly destabilizing (substituting residues—alanine, serine, cysteine) or (2) highly destabilizing (substituting residues—arginine, valine, glutamic acid, aspartic acid, tryptophan) [22]. If a patient presented multiple variants affecting one or multiple COL4 genes, variant classification was performed only for the mutation with the highest pathogenicity.

### 2.4. Statistical Analysis

Continuous variables were expressed as mean (±standard deviation [SD]) or median (interquartile range [IQR]) according to their distribution. Categorial data were presented as frequencies and percentages. When comparing continuous variables, parametric (Student’s t tests and one-way ANOVAs) and nonparametric tests (Mann–Whitney U tests and Kruskal–Wallis tests) were used depending on each variable distribution. Categorical data were compared using chi-squared tests. Renal survival was analyzed using the Kaplan–Meier method, and log-rank tests were used for comparison. The date of birth was assigned as the starting point. The end point was considered the age at the time of ESKD onset or at the last observation available. In the subgroups where more than half of the subjects developed ESKD by the end of the observation period, the results were reported as median renal survival time (with confidence interval [95% CI]). Otherwise, the results were reported as mean renal survival time (with 95% CI). Hazard ratios (with 95% CI) were calculated using a univariate Cox regression model.

Statistical analysis was performed using IBM SPSS Statistics software (version 29.0.1.0). The figures were generated using GraphPad Prism software (version 10.1.1). The level of significance was selected at 0.05 (two-sided).

### 2.5. Ethics Approval and Consent to Participate

The study was approved by the Ethics Committee of the Fundeni Clinical Institute (approval number: 66034, date: 15 December 2023). All subjects provided written informed consent for participation. All the research was conducted according to the guidelines of the Declaration of Helsinki.

## 3. Results

### 3.1. Study Population

Among the 36 patients, the median age at clinical onset was 33 years (IQR, 23–42). There were 17 females (47.2%), with all the subjects being Caucasian. Only seven patients were related from three distinct families.

### 3.2. Clinical Features

**Figure 1:**
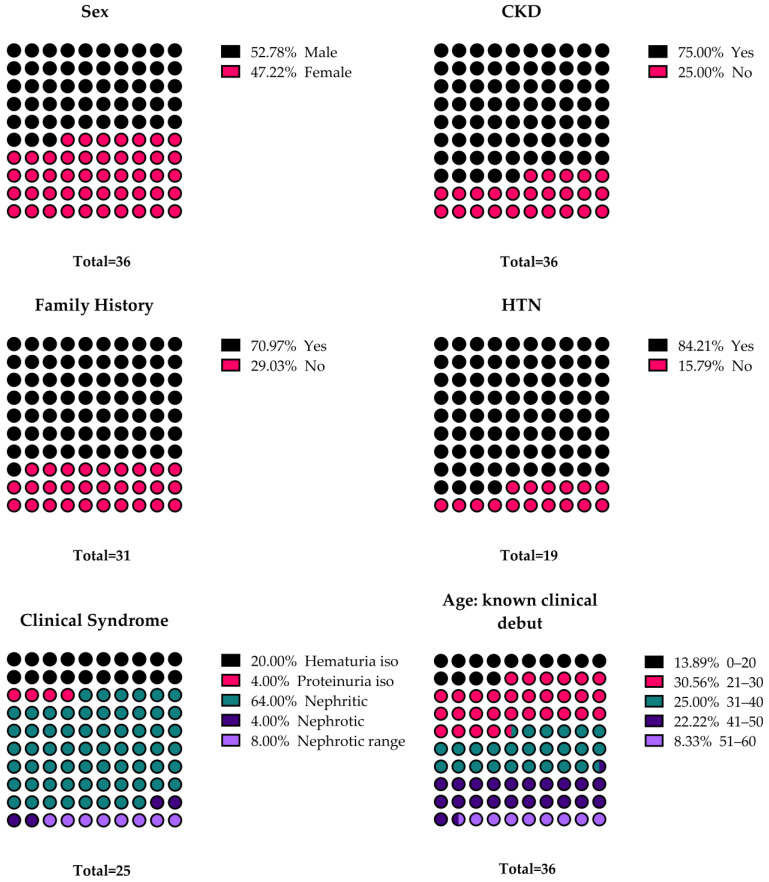
The proportions for each demographic and clinical characteristic of patients included in our cohort. Results are illustrated as percentages. The total number of cases for which the data were available is depicted under each diagram. CKD—chronic kidney disease; Hematuria iso—isolated hematuria; HTN—hypertension; Nephritic—nephritic syndrome; Nephrotic—nephrotic syndrome; Nephrotic range—Nephrotic range proteinuria; Proteinuria iso—isolated proteinuria.

Most of the patients had a family history of kidney disease (71%). The most prevalent clinical picture before referral was nephritic syndrome (64%), followed by isolated hematuria (20%), nephrotic range proteinuria (8%), isolated proteinuria (4%), and overt nephrotic syndrome (4%). A total of 75% of patients had CKD at the time of genetic testing. More than one-third of the patients screened for extrarenal manifestations had hearing loss (28.6%) and eye abnormalities (7.1%). In the subgroup of patients with hearing abnormalities there were three subjects with XLAS, four with ADAS, and one with ARAS. The general characteristics of the cohort are illustrated in Figure 1.

### 3.3. Pathological Findings

A kidney biopsy was performed in 36.1% of the patients. Between each kidney biopsy and genetic testing there was a median delay of 61.5 months (IQR, 16.75–92.25). The most common findings in the pathology reports were structural alterations of the GBM suggestive of AS or TBM disease (61.6%), with the rest of the patients having FSGS (23.1%), vascular lesions (7.7%), or other nonspecific lesions such as global glomerulosclerosis or interstitial fibrosis (7.7%). EM data were available for six patients, of which 83.3% had podocyte foot process effacement (three focal and two diffuse). There were no significant differences regarding proteinuria for those with FSGS (2.26 ± 1.86 g/day compared to 2.43 ± 1.63 g/day, *p* = 0.98). The FSGS group displayed a mean eGFR of 41 ± 29.5 mL/min/1.73 m^2^, which was lower when compared to the rest of the cohort (54.5 ± 45.2 mL/min/1.73 m^2^, *p* = 0.18).

### 3.4. Laboratory Findings

At the time of referral to our center, patients had a mean serum creatinine of 3.83 ± 3.51 mg/dL, corresponding to a mean eGFR of 46.2 ± 39 mL/min/1.73 m^2^. The distribution regarding CKD stages was as follows: stage G1—8.57%, stage G2—28.57%, stage G3a—8.57%, stage G3b—5.71%, stage G4—11.42%, and stage G5—37.14%.

Hematuria was present in 76.92% of cases, of which only one subject had macroscopic hematuria. Patients without hematuria had a mean eGFR of 42 ± 30.9 mL/min/1.73 m^2^ compared to 57.6 + 42.3 mL/min/1.73 m^2^ for those who exhibited hematuria (*p* = 0.18).

Data on proteinuria were available for 41.7% of patients, of whom 73.3% had proteinuria of over 1 g/day. Subjects with proteinuria had a significantly lower mean eGFR when compared to those without proteinuria (38.27 ± 30.45 mL/min/1.73 m^2^ compared to 90.25 ± 32.98 mL/min/1.73 m^2^; *p* = 0.026). For the whole cohort, the mean proteinuria was 2.04 ± 1.53 g/day.

Excluding the patients who already had ESKD at referral (41.6%), our subjects had a mean eGFR of 65.9 ± 35.2 mL/min/1.73 m^2^ and a mean proteinuria of 1.77 ± 1.44 g/day. Seventy-five percent of this group of patients had hematuria. Regarding proteinuria, we observed higher values for those with ESKD (3.13 ± 1.68 g/day compared to 1.77 ± 1.44 g/day; *p* = 0.82). The laboratory findings at referral are detailed in Table 1.

### 3.5. Treatment History

Regarding treatment before genetic testing, data were available for 66.7% of patients, of whom 70.8% received RAASis, 29.2% received immunosuppression, and 8.3% received SGLT2is. The mean eGFR for those receiving immunosuppression was 42.86 ± 33.39 mL/min/1.73 m^2^, compared to 47.94 ± 40.77 mL/min/1.73 m^2^ for those without immunosuppression (*p* > 0.99). Mean proteinuria was higher in the immunosuppression group (2.3 ± 1.4 g/day compared to 1.8 ± 1.6 g/day; *p* = 0.34).

### 3.6. Genetic Testing

The median age at the time of genetic testing was 39.5 years (IQR, 27.5–48.25). The median period between the known clinical onset and genetic testing was 4 years (IQR, 0–8.35).

A total of 24 different variants were identified, of which 10 had not been described in the literature according to the laboratory reports at that time. Up to December 2023, eight of those variants remain undescribed. Four variants were repeated more than once in nonrelated subjects. The characteristics of each variant are detailed in Table 2.

Mutations affecting each of the COL4 α chains were equally prevalent (33.3%). Six patients had multiple variants involving COL4 genes. The genetic and clinical features of the patients included in this group are illustrated in Table S1.

A total of 61.1% of variants were classified as PV, 22.2% were considered LPV, and only 16.7% were classified as VUS. The genetic and clinical features of patients with variants classified as VUS are illustrated in Table S2.

Regarding zygosity, 5.6% of variants were homozygous, 72.2% were heterozygous and 22.2% were hemizygous. Out of the 26 heterozygous variants, there were four females with variants involving the α 5 chain of the COL4 molecule (COL4A5). A total of 33.3% of cases showed X-linked inheritance, whereas 63.9% were autosomal forms (of which 91.31% were dominant and 8.69% were recessive). One patient had three variants involving the COL4A3, COL4A4, and COL4A5 genes simultaneously. He was classified as having a complex pattern of inheritance.

Regarding the pathogenicity of the variants depending on the gene involved, there were 5 PV, 5 LPV, and 2 VUS in the COL4A3 group, 7 PV, 1 LPV, and 4 VUS in the COL4A4 group, and 10 PV and 2 LPV in the COL4A5 group. Out of the four females with COL4A5 variants, three had a PV and one had an LPV.

**Figure 2:**
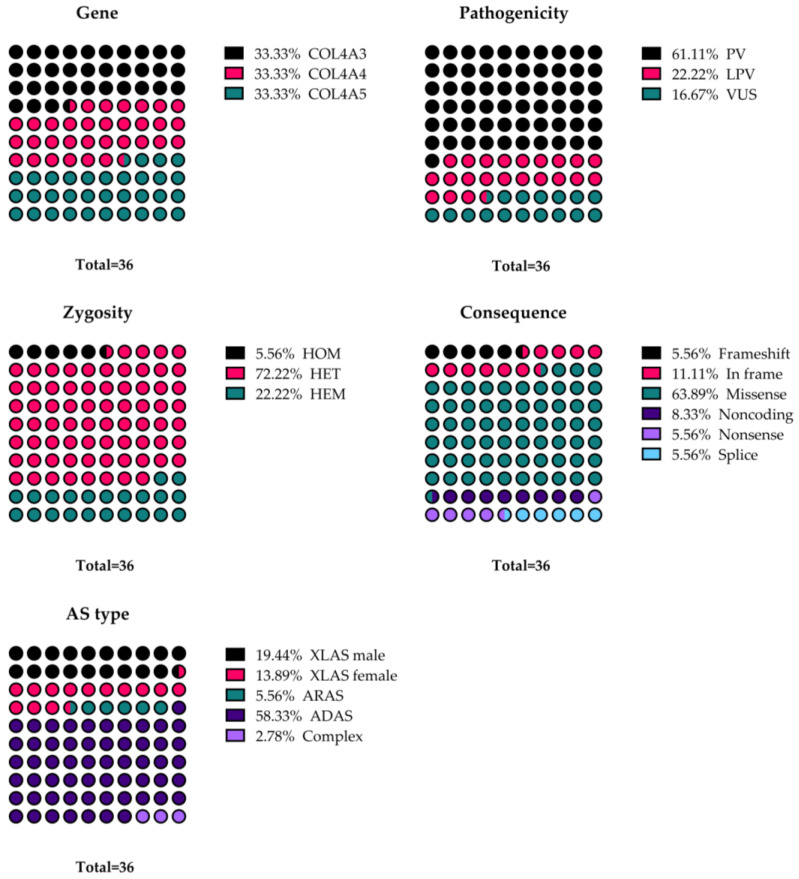
The proportions for each characteristic of type IV collagen variants in our cohort. Results are illustrated as percentages. The total number of cases is illustrated under each diagram. ADAS—autosomal dominant Alport syndrome; ARAS—autosomal recessive Alport syndrome; COL4A3—the α 3 chain of type IV collagen molecule; COL4A4—the α 3 chain of type IV collagen molecule; COL4A5—the α 5 chain of type IV collagen molecule; Complex—Alport syndrome with complex inheritance; Frameshift—frameshift variants; HEM—hemizygous; HET—heterozygous; HOM—homozygous; In frame—in frame deletion and insertion variants; LPV—likely pathogenic variant; Missense—missense variants; Noncoding—noncoding variants; Nonsense—nonsense variants; PV—pathogenic variant; Splice—splice junction loss variants; VUS—variants of uncertain significance; XLAS—X-linked Alport syndrome.

There were 63.9% missense variants, 11.1% in frame variants, 8.3% noncoding variants, 5.6% nonsense variants, 5.6% frameshift variants, and 5.6% splice junction loss variants. The proportions of these genetic characteristics are depicted in Figure 2.

In 82.6% of missense variants, glycine was the substituted amino acid. A total of 68.4% of glycine substitutions involved a highly destabilizing residue. When classifying variants according to exon locations, 91.3% of the substitutions were located between exon 21 and the carboxyl-terminus.

Three families comprising seven subjects were included. Related patients had similar renal function at presentation (all being in the same CKD category), except for one male with X-linked AS who had ESKD compared to his female siblings who displayed normal renal function. There were four variants that repeated more than once in nonrelated subjects.

There were no significant differences regarding the laboratory findings at diagnosis between patients with COL4A3, COL4A4, and COL4A5 mutations (Table 3). Although most of the patients with ESKD had a COL4A3 variant (44.4%), the proportion of ESKD patients did not differ significantly among the three groups (*p* = 0.25). There were no differences regarding the median ESKD onset age (*p* = 0.51). The COL4A3 group displayed a lower mean GFR of 40.42 ± 41.36 mL/min/1.73 m^2^ (*p* = 0.69), owing to the sizable proportion of patients presenting with ESKD. The comparison between the characteristics of the three groups is summarized in Table 3.

There were no differences in terms of renal function and proteinuria when comparing missense with non-missense variants (*p* = 0.64 and *p* = 0.06, respectively). All the patients with non-missense mutation presented with hematuria compared to only 64.7% of the patients with missense variants.

### 3.7. Renal Survival

There were 15 patients (41.6%) who presented with ESKD and three patients (8.3%) who later developed ESKD. For those with ESKD, the median age at ESKD diagnosis was 25.5 years (IQR, 21.5–37.25). A total of 61.1% of those with ESKD underwent kidney transplantation at a median age of 27 years (IQR, 24–42). Most of the subjects with ESKD had COL4A3 variants (44.4%) followed by COL4A5 (33.3%) and COL4A4 (22.2%). As shown above in Table 3, patients with COL4A4 variants displayed a higher median age at the time of ESKD onset.

The overall median kidney survival was 42 years (95% CI, 29.98–54.01), meaning that half of all patients presented with ESKD by the age of 42. The COL4A4 group displayed significantly better renal survival than the COL4A3 group (*p* = 0.027). There was a nearly 70% reduction in the risk of ESKD when comparing the COL4A3 to COL4A4 variants (HR, 0.304 [95% CI, 0.9–1.03]; *p* = 0.056). Although patients with heterozygous mutations displayed a higher mean renal survival time than those with homozygous and hemizygous forms, the differences were not significant (*p* = 0.052 and *p* = 0.053, respectively; see Table 4).

Regarding the type of AS, there were no significant differences (*p* = 0.29; see Table 4). Patients with ADAS displayed the best renal prognosis, with a mean kidney survival of 42.48 years (95% CI, 36.08–48.88; see Table 4). Although only one female who was heterozygous for a COL4A5 variant developed ESKD, the difference in kidney survival between females and males with XLAS was not significant (*p* = 0.22).

Only 40.9% of patients with missense variants developed ESKD compared to 69.23% of those with other types of mutations (*p* = 0.084). Missense variants displayed significantly better renal survival compared to all the other types of mutations (*p* = 0.023; see Table 4). There was a 66% reduction in the risk of ESKD in patients with missense variants (HR, 0.33 [95% CI, 0.12–0.9]; *p* = 0.031).

Although patients with VUS had higher renal survival compared to those with PV and LPV, the differences were not significant (*p* = 0.81; detailed in Tabel 4). Furthermore, when comparing the renal survival of patients with LP and LPV together to that of those with VUS, the difference did not achieve statistical significance (39.06 years [95% CI, 33.74–44.37] compared to 43.66 years [95% CI, 33.8–53.52]; *p* = 0.57). Other characteristics, such as inheritance, had no significant influence on renal survival (*p* = 0.89). The influence of variant characteristics on kidney survival is detailed in Table 4.

As presented in Table 5, there were no differences in renal survival depending on the substituted amino acid, the substituting residue, and the molecular location of the substitution in missense variants.

**Figure 3:**
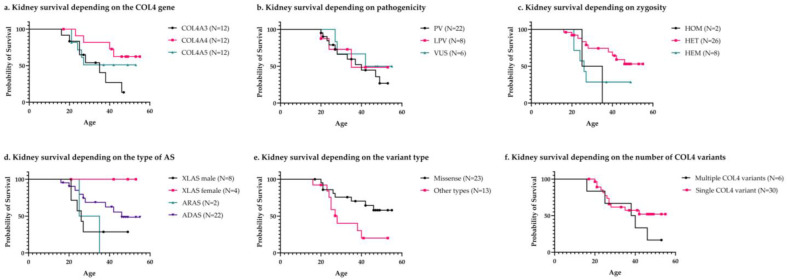
Kaplan–Meier curves comparing kidney survival depending on the characteristics of type IV collagen variants. Kidney survival is depicted depending on the following: (**a**) gene involved, (**b**) pathogenicity, (**c**) zygosity, (**d**) type of Alport syndrome, (**e**) coding impact, and (**f**) number of variants involving type IV collagen genes. As there is only one patient with a complex pattern of inheritance, data regarding his renal survival are not illustrated in panel d. Censored cases are represented by symbols (circle, square, triangle, and diamond). The number of cases is specified in the legend in brackets as “N=”. ADAS—autosomal dominant Alport syndrome; ARAS—autosomal recessive Alport syndrome; COL4A3—the α 3 chain of type IV collagen molecule; COL4A4—the α 3 chain of type IV collagen molecule; COL4A5—the α 5 chain of type IV collagen molecule; HEM—hemizygous; HET—heterozygous; HOM—homozygous; LPV—likely pathogenic variants; Missense—missense variants; PV—pathogenic variants; VUS—variants of uncertain significance; XLAS—X-linked Alport syndrome.

The Kaplan–Meier curves comparing kidney survival depending on the characteristics of the variants are depicted in Figure 3. Males had a lower mean renal survival of only 36.36 years (95% CI, 29.85–42.87) compared to 43.96 years (95% CI, 36.8–49.98) for females (*p* = 0.11). There were twice as many males with ESKD compared to females (12 compared to six, *p* = 0.09).

All eight participants with clinically significant hearing loss developed ESKD. The median renal survival in this group was 25 years (95% CI, 23.65–26.34), which was significantly lower than that in those with no hearing abnormality (*p* < 0.001). Hearing loss was associated with a 10-fold increase in the risk of ESKD (HR, 10.91 [95% CI, 3.16–37.7]; *p* < 0.001). There were no correlations between the characteristics of COL4 variants (i.e., gene, inheritance, pathogenicity, and mutation consequences) and the risk of having hearing abnormalities.

None of the patients with proteinuria under 1 g/day developed ESKD, compared to 54.54% of those with proteinuria greater than 1 g/day (*p* = 0.1). Median renal survival was only 27 years (95% CI, 2.06–29.94) for those with nephrotic range proteinuria (*p* = 0.1). Patients with hematuria had a lower mean renal survival time (41.2 years [95% CI, 34.88–47.52] compared to 50.16 years [95% CI, 41.51–58.81]; *p* = 0.21).

## 4. Discussion

The present study examines the natural history of patients with AS and the relationship between the characteristics of the variant and kidney survival. In our diverse cohort of 36 patients diagnosed with AS through genetic testing, we identified that subjects with COL4A4 variants and those with missense variants had significantly better kidney survival, whereas those with COL4A3 variants and those with clinically significant hearing loss had worse renal prognoses.

Owing to the large phenotypic heterogenicity and nonspecific histological lesions, current guidelines recommend genetic testing as a first-line diagnostic test whenever there are clinical, histological or pedigree data suggestive of AS [7,35]. In our cohort, there was an important delay in the referral of the patients, with the median period between clinical onset and sampling for genetic testing being 4 years (IQR, 0–8.35). Therefore, our cohort included 15 subjects (41.66%) who presented in our clinic with ESKD of unknown etiology, which is consistent with data reported in the literature suggesting that more than 60% of patients with CKD and COL4 variants do not have a clinical diagnosis of AS before their genetic tests.

In the subgroup of patients who underwent kidney biopsies, only 60% had lesions on light or electron microscopy (LM or EM) suggestive of AS or TBM disease, which is in accordance with data reported in the literature regarding the difficulties of histologic diagnosis of AS, especially in females with XLAS and in autosomal forms [6]. Considering that AS can cause histologic lesions of FSGS that, in the absence of genetic testing, can be interpreted as a primary podocytopathy [36,37], we identified three patients with histologically diagnosed FSGS in our cohort. All patients in this subgroup received immunosuppression before the diagnosis of COL4-related nephropathy. Nearly 30% of all our cohort received some sort of immunosuppression before the diagnosis of AS. Although not statistically significant, this group displayed higher values of proteinuria.

Regarding the results of genetic testing, we identified 24 different variants involving the COL4 genes. Only seven patients were related, comprising three distinct families. Our cohort included mostly variants inherited in an autosomal dominant manner (58.33%), possibly causing the delay in genetic testing because autosomal dominant forms tend to have nonspecific and variable clinical pictures with slower declines in renal function [16]. Our high proportion of ADAS is supported by recent data suggesting a higher frequency of these forms than previously considered, with prevalence largely ranging between 15 and 30% of AS cases [16,38]. Due to the limited number of patients, the difference between females and males with XLAS was not significant, although most men had PV and developed ESKD compared to women who had better kidney survival.

Although current guidelines recommend considering only PV and LPV as disease causing [7,35], we included in our analysis six subjects with variants classified as VUS, as they had clinical and/or histological features suggestive of AS and/or FSGS.

Missense variants are well known for being the most frequent type of mutation regardless of the COL4 gene involved, the vast majority being represented by glycine substitutions. Accordingly, 63.9% of variants analyzed were missense mutations, with 82.6% of them involving glycine as the substituted residue.

Hematuria was the most prevalent clinical finding, with 76.92% of patients having hematuria at the time of first evaluation, and nearly 84% having a personal history of hematuria before referral to our center. A total of 73.33% of our patients had proteinuria greater than 1 g/day, with more than a third of them having nephrotic range proteinuria (36.36%). We observed a significant impact on renal function, with lower eGFR in the group with proteinuria greater than 1 g/day. None of the patients with proteinuria lower than 1 g/day developed ESKD, so comparing renal survival in the two groups was not possible. In a large retrospective study, Furlano et al. observed a five-fold increase in the risk of KRT in the presence of proteinuria [16], and while our results did not reach statistical significance, we observed a lower renal survival in this group of patients compared to the rest of the cohort.

The natural history of AS has been extensively studied, with men hemizygous for COL4A5 mutations having a worse renal prognosis, 90% of them being on KRT by the age of 40, and females heterozygous for COL4A5 mutations having milder outcomes, with only 30% of them having ESKD by the age of 60 [5,39]. In the case of the autosomal dominant forms, the prognosis is much better, with the reported median renal survival time being above 60 years [16]. Both males and females with ARAS will develop ESKD by the age of 40.

Although our cohort was comprised mostly of ADAS, considered in the literature as having a milder phenotype [5,16], the median renal survival observed in our study was 42 years (95% CI, 29.98–54.01). In line with these findings, autosomal inherited variants also displayed a worse evolution than expected with a mean renal survival time of only 41.15 years (95% CI, 35.08–47.21). Overall, half of the patients presented with or later developed ESKD, at a median age of onset of 25.5 years (IQR, 21.5–37.25), similar to the one seen in men with XLAS with mutations with an important molecular impact, such as large deletions [40].

Consistent with the results of Ozdemir et al., who observed a faster progression to CKD in children with COL4A3 mutations [41], in our cohort, patients with COL4A3 variants displayed poorer prognoses, with most patients with ESKD coming from this group. The lowest renal survival time was observed in this group. Better outcomes were observed in those with COL4A4 variants, who had the highest renal survival and the highest median age at the time of ESKD diagnosis. One third (4/12) of the patients with COL4A4 variants were classified as VUS, in comparison with only one sixth (2/12) of the patients with COL4A3, explaining the difference in renal survival between the two groups. Furthermore, although 50% (2/4) of the patients with VUS involving COL4A4 developed ESKD, the age of onset was higher than the mean renal survival observed in the whole cohort (42 and 57 years, respectively; refer to Table S2). There was a nonsignificant tendency toward better renal survival in those with heterozygous mutations, representing the patients with ADAS and the females with XLAS. Since there were no disparities in renal survival between VUS, PV, and LPV, we believe that the significant number of VUS patients developing ESKD (3/6, refer to Table S2) has influenced renal survival.

One of the features included in the classical description of AS, bilateral sensorineural hearing loss, usually develops progressively, affecting most males with XLAS by the age of 40 [5]. In our cohort, only eight out of the 28 subjects screened exhibited hearing loss (28.6%), but the real prevalence might be higher as our results were based on medical records and audiometry data were not available. Consistent with data reported in the literature on XLAS [42,43], hearing loss was associated with a poorer renal outcome in our study, as all eight patients had ESKD with a median renal survival of only 25 years (95% CI, 23.65–26.34; *p* < 0.001).

Although there is much debate regarding the mechanism of hearing loss in AS [44,45], there are studies reporting correlations between the molecular characteristics of COL4 variants and the risk of hearing abnormalities, especially regarding the type of substituting residue in missense variants [15]. Our study failed to identify associations between the characteristics of the variants (i.e., gene, inheritance, pathogenicity, and type of mutations) and the risk of developing sensorineural hearing loss. There were only two patients with eye abnormalities consistent with AS in our cohort, owing to the limited ophthalmological screening.

Although missense variants are associated with a less severe phenotype in all forms of AS [12], there is a high degree of variability regarding the renal prognosis depending on the characteristics of the variant, such as the substituting residue and the position of the substitution [15]. In our study, we managed to replicate some of these findings, with missense variants displaying a nearly 12-year increase in the mean kidney survival and a 66% reduction in the risk of developing ESKD. The characteristics of the substituting residue and the position of the substitution did not impact the renal outcomes, probably because most of the variants involved destabilizing amino acids as substituting residues and were located between exon 21 and the carboxyl terminal domain, thus already possessing a higher risk of kidney dysfunction.

One of the most important limitations of this study is the relatively small size and the monocentric nature of the cohort. Data regarding proteinuria were incomplete due to the retrospective design and the use of medical records for data collection. As audiometric reports were not available, the exact prevalence of hearing loss might be higher than reported in our study.

The strength of our study comes from the diversity of our cohort, comprising patients with different forms of AS, different types of mutations, and covering the entire clinical spectrum of COL4-related diseases. As there was a high proportion of ADAS in our cohort with lower renal survival than reported in the literature, we were able to argue that even the classically milder forms of AS can be at risk of rapid progression to ESKD.

## 5. Conclusions

In summary, our study has identified that the type of variant and the α chain involved are key factors to consider when assessing the prognosis in AS. As autosomal dominant forms and missense variants, classically considered as having a milder phenotype, displayed worse kidney survival than expected, our research highlights the need for refining individual prognostication based on the molecular characteristics of each variant. Our diverse cohort illustrated the wide phenotypic spectrum of AS, which is probably responsible for the delay in diagnosis.

## Figures and Tables

**Table 1:** Laboratory findings at referral.

Laboratory Findings at Referral			
Including Patients Presenting with ESKD		Excluding Patients Presenting with ESKD	
Serum Creatinine, mg/dL			
Mean ± SD	3.83 ± 3.51	Mean ± SD	1.56 ± 0.87
No./total (%)	36/36 (100)	No./total (%)	21/21 (100)
eGFR, mL/min per 1.73 m^2^			
Mean ± SD	46.2 ± 39.01	Mean ± SD	65.95 ± 35.28
No./total (%)	36/36 (100)	No./total (%)	21/21 (100)
Proteinuria, g/day			
Mean ± SD	2.04 ± 1.53	Mean ± SD	1.77 ± 1.44
No./total (%)	15/36 (41.7)	No./total (%)	12/21 (57.1)
Hematuria, no./total (%)			
Hematuria	20/26 (76.9)	Hematuria	15/20 (75)
No./total (%)	26/36 (72.2)	No./total (%)	20/21 (95.2)

Illustrated data include the entire cohort as well as the patients without end-stage kidney disease. eGFR—estimated glomerular filtration rate; ESKD—end-stage kidney disease; SD—standard deviation.

**Table 2:** Characteristics of the 24 different variants of type IV collagen genes.

Patient Number	Variant Characteristics								Type of AS	Previously Described
	Gene	Position	Nucleotide Change	Amino Acid Change	Zygosity	Pathogenicity	Consequence	Inheritance		
33	COL4A3	Exon 26	c.1814G>T	p.(Gly605Val)	HET	LPV	Missense	AD, AR	ADAS	No *
34	COL4A3	Exon 26	c.1855G>A	p.(Gly619Arg)	HET	PV	Missense	AD, AR	ADAS	Yes [23]
15	COL4A3	Exon 32	c.2549G>A	p.(Gly850Glu)	HET	LPV	Missense	AD, AR	ADAS	Yes [24]
29	COL4A3	Intron 14	c.2746+1G>T	p.?	HET	PV	Noncoding	AD, AR	ADAS	Yes [25]
8, 25	COL4A3	Exon 38	c.3321_3329del	p.(Ser1108_Gly1110del)	HET	LPV	In frame	AD, AR	ADAS	Yes [26]
19	COL4A3	Exon 41	c.3546_3548dup	p.(Gly1183dup)	HET	VUS	In frame	AD, AR	ADAS	No *
1	COL4A3	Exon 42	c.3602G>A	p.(Gly1201Asp)	HOM	LPV	Missense	AD, AR	ARAS	No *
26	COL4A3	Exon 44	c.3925C>T	p.(Pro1309Ser)	HET	VUS	Missense	AD, AR	ADAS	No *
6	COL4A3	Exon 1	c.40_63del	p.(Leu14_Leu21del)	HET	PV	In frame	AD, AR	ADAS	Yes [27]
10	COL4A3	Exon 48	c.4348C>T	p.(Arg1450*)	HET	PV	Nonsense	AD, AR	ADAS	Yes [28]
2	COL4A3	Exon 51	c.4825C>T	p.(Arg1609*)	HOM	PV	Nonsense	AD, AR	ARAS	Yes [28]
22, 32	COL4A4	Exon 20	c.1321_1369+3del	52bp-Deletion	HET	PV	Splice junction loss	AD, AR	ADAS	Yes [29]
28	COL4A4	Exon 24	c.1716del	p.(Pro573Leufs*80)	HET	PV	Frameshift	AD, AR	ADAS	Yes [29]
3	COL4A4	Exon 27	c.2159C>T	p.(Pro720Leu)	HET	VUS	Missense	AD, AR	ADAS	No *
17, 20, 30, 31	COL4A4	Exon 31	c.2734G>C	p.(Gly912Arg)	HET	LPV/PV **	Missense	AD, AR	ADAS	No *
36	COL4A4	Exon 41	c.3961del	p.(Asp1321Metfs*67)	HET	PV	Frameshift	Complex inheritance	Complex	Yes [29]
23, 27	COL4A4	Exon 48	c.5045G>A	p.(Arg1682Gln)	HET	VUS	Missense	AD, AR	ADAS	Yes [30]
35	COL4A4	Intron 14	c.871-3A>G	p.?	HET	VUS	Noncoding	AD, AR	ADAS	No *
9	COL4A5	Exon 20	c.1226G>A	p.(Gly409Asp)	HEM	PV	Missense	X-linked	X-linked male	Yes [31]
14, 16	COL4A5	Exon 25	c.1871G>A	p.(Gly624Asp)	HEM	PV	Missense	X-linked	X-linked male	Yes [32]
24	COL4A5	Exon 31	c.2605G>A	p.(Gly869Arg)	HEM	PV	Missense	X-linked	X-linked male	Yes [31]
4, 5, 7, 11, 12, 13	COL4A5	Exon 41	c.3721G>T	p.(Gly1241Cys)	HET/HEM	PV	Missense	X-linked	X-linked male/female	Yes [14]
18	COL4A5	Exon 11	c.637G>C	p.(Gly213Arg)	HET	LPV	Missense	X-linked	X-linked female	Yes [31]
21	COL4A5	Intron 12	c.688-1G>A	p.?	HEM	LPV	Noncoding	X-linked	X-linked male	No *

* VarSome tools [18], ClinVar database [33] and LOVD v.3.0 database [34] were used to assess each variant. ** In patient nos. 20, 30 and 31 the variant was assessed by the testing laboratory as being a pathogenic variant, whereas in patient no. 17 it was classified by a different laboratory as being a likely pathogenic variant. ADAS—autosomal dominant Alport syndrome; ARAS—autosomal recessive Alport syndrome; COL4A3—the α 3 chain of type IV collagen molecule; COL4A4—the α 3 chain of type IV collagen molecule; COL4A5—the α 5 chain of type IV collagen molecule; Complex—Alport syndrome with complex inheritance; Frameshift—frameshift variants; HEM—hemizygous; HET—heterozygous; HOM—homozygous; In frame—in frame deletion and insertion variants; LPV—likely pathogenic variant; Missense—missense variants; Noncoding—noncoding variants; Nonsense—nonsense variants; PV—pathogenic variant; Splice—splice junction loss variants; VUS—variants of uncertain significance; X-linked—X-linked Alport syndrome.

**Table 3:** Differences in clinical features and laboratory findings depending on the gene involved.

Characteristics	Type of Collagen α Chain			*p* Value
	COL4A3	COL4A4	COL4A5	
Sex, No. (%)				
Male	4/36 (11.1)	7/36 (19.4)	8/36 (22.2)	0.50
Female	8/36 (22.2)	5/36 (13.8)	4/36 (11.1)	0.46
Laboratory findings				
Serum creatinine, mg/dL	4.12 ± 3.34	3.75 ± 3.99	3.78 ± 3.46	0.82
eGFR, mL/min per 1.73 m^2^	40.42 ± 41.36	48.33 ± 33.14	50.18 ± 44.94	0.69
Proteinuria, g/day	2.2 ± 1.64	2.12 ± 1.69	1.28 ± 1.01	0.77
Hematuria, no./total (%)	6/7 (85.7)	8/10 (80)	6/9 (66.7)	0.64
ESKD, no./total (%)				
ESKD at diagnosis	7/12 (58.3)	3/12 (25)	5/12 (41.6)	0.25
Progression to ESKD	1/12 (8.3)	1/12 (8.3)	1/12 (8.3)	1
Kidney transplant	6/12 (50)	1/12 (8.3)	4/12 (33.3)	0.08
Age, median (IQR), y.				
ESKD	25 (20–35)	41 (27.25–53.25)	24 (21–26.5)	0.51
Kidney transplant	27 (22.75–38)	-	25.5 (23.25–39.75)	0.38

COL4A3—the α 3 chain of type IV collagen molecule; COL4A4—the α 3 chain of type IV collagen molecule; COL4A5—the α 5 chain of type IV collagen molecule; CKD—chronic kidney disease; eGFR—estimated glomerular filtration rate; ESKD—end-stage kidney disease; IQR—interquartile range; y.—years.

**Table 4:** The mean renal survival depending on the characteristics of the variant.

**Mean kidney survival (95% CI), y.**	Type of collagen α chain										*p* value
	COL4A3		COL4A4			COL4A5			Overall		0.12
	33.06 (22.79–39.34)		46.83 (39.89–53.76)			38.79 (29.79–47.79)			40.55 (35.55–45.55)		
	Inheritance										0.891
	X-linked		Autosomal			Digenic/Complex			Overall		
	38.79 (29.79–47.98)		41.15 (35.08–47.21)			40 (40–40)			40.55 (35.55–45.55)		
	Zygosity										0.054
	Homozygous		Heterozygous			Hemizygous			Overall		
	30 (20.2–39.8)		44.07 (38.55–49.6)			31 (22.42–39.57)			40.55 (35.55–45.55)		
	Classification										0.81
	Pathogenic		Likely Pathogenic			VUS			Overall		
	38.51 (32.32–44.72)		40.27 (29.63–50.91)			43.66 (33.80–53.52)			40.55 (35.55–45.55)		
	Multiple mutations involving COL4 genes										0.23
	Yes			No				Overall			
	36.33 (26.37–46.29)			41.67 (35.99–47.34)				40.55 (35.55–45.55)			
	Coding impact										0.023 *
	Missense			Another type				Overall			
	44.8 (38.87–50.72)			32.98 (26.05–39.91)				40.55 (35.55–45.55)			
	Type of AS										0.29
	XLAS males		XLAS females		ARAS		ADAS		Complex		
	32.16 (22.47–41.86)		45.75 (33.44–58.05)		30 (20.2–39.8)		42.48 (36.08–48.88)		40 (40–40)		

Significant differences are depicted using “*” sign. ADAS—autosomal dominant Alport syndrome; ARAS—autosomal recessive Alport syndrome; AS—Alport syndrome; COL4—type IV collagen; XLAS α 3 chain of type IV collagen molecule; COL4A4—the α 3 chain of type IV collagen molecule; COL4A5- the α 5 chain of type IV collagen molecule; CI—confidence interval; Missense—missense variants; VUS—variants of uncertain significance; XLAS—X-linked Alport syndrome; y.—years.

**Table 5:** Mean renal survival for patients with missense variants depending on the molecular characteristics.

**Mean kidney survival (95% CI), y.**	Substituted residue (all missense variants)			*p* value
	Glycine	Another amino acid	Overall	0.41
	42.05 (35.49–48.62)	51.75 (46.23–57.26)	44.8 (38.87–50.72)	
	Substituting residue (glycine substitutions)			0.92
	Destabilizing residues (Arg/Val/Glu/Asp/Trp)	Non-destabilizing residues (Ala/Cys/Ser)	Overall	
	42.72 (35.26–50.18)	39.66 (25.88–53.45)	42.05 (35.49–48.62)	
	Molecular location (all missense variants)			0.55
	Exon 1–20	Exon 21-carboxi terminus	Overall	
	31.5 (16.94–46.05)	45.53 (39.51–51.51)	44.8 (38.87–50.72)	

Ala—alanine; Arg—arginine; Asp—aspartic acid; CI—confidence interval; Cys—cysteine; Glu—glutamic acid; Ser—serine; Trp—tryptophan; Val—valine; y.—years.

## Data Availability

The datasets used during the current study are available from the corresponding author on reasonable request. The data are not publicly available due to privacy restrictions.

## Supplementary Materials

The following supporting information can be downloaded at https://www.mdpi.com/article/10.3390/genes15050593/s1: Table S1: Genetic and clinical characteristics of patients with multiple type IV collagen variants; Table S2: Genetic and clinical characteristics of patients having a variant of uncertain significance.
