# Supplementary material for: Genotype–Phenotype Correlations in Alport Syndrome—A Single-Center Experience
Source: Genes (Basel). 2024 May 7;15(5):593. doi: 10.3390/genes15050593 (PMC11121304; doi:10.3390/genes15050593)
Supplement: Supplementary file 1 [file genes-15-00593-s001.zip › genes-2988516-supplementary.pdf]

SUPPLEMENTARY MATERIAL

Table S1. Genetic and clinical characteristics of patients with multiple type IV collagen variants

Variant no. 3		Laboratory findings at referral				Outcomes		
Consequence	Inertance	Serum creatinine (mg/dl)	eGFR (ml/min/1.73 m2)	Proteinuria (g/day)	Hematuria	ESKD	Age ESKD (y.)	Kidney transplant
NA	NA	8.9	7	NA	Absent	Yes (referral)	38	No
NA	NA	1	80	NA	Absent	Yes (referral)	16	Yes (16 y.)
NA	NA	2.54	22	2.9	Microscopic	No	NA	No
NA	NA	1.36	54	NA	Absent	Yes (referral)	25	Yes (26 y.)
NA	NA	3.62	15	1.14	Microscopic	Yes (follow-up)	46	No
In frame	AD, AR	8.36	8	NA	Absent	Yes (referral)	40	No

				Variant no. 2					
Zygosity	Pathogenicity	Consequence	Inheritance	Gene	Position (Exon)	Nucleotide change	Amino acid change	Zygosity	Pathogenicity
HET	LPV	Missense	AD, AR	NA	NA	NA	NA	NA	NA
HET	PV	Frame shift	AD, AR	NA	NA	NA	NA	NA	NA
HET	VUS	Missense	AD, AR	NA	NA	NA	NA	NA	NA
HET	VUS	Non-coding	AD, AR	NA	NA	NA	NA	NA	NA
HET	LPV	Missense	AD, AR	NA	NA	NA	NA	NA	NA
HEM	LPV	Missense	X-linked	COL4A3	41	c.3546_3548dup	p.(Gly1183dup)	HET	VUS

		Variant no. 1							
Nucleotide change	Amino acid change	Zygosity	Pathogenicity	Consequence	Inheritance	Gene	Position (Exon)	Nucleotide change	Amino acid change
c.40_63del	p.(Leu14_Leu21del)	HET	PV	In frame	AD, AR	COL4A3	26	c.1814G>T	p.(Gly605Val)
c.4348C>T	p.(Arg1450*)	HET	PV	Non-sense	AD, AR	COL4A3	25	c.1716del	p.(Pro573Leufs*80)
c.2734G>C	p.(Gly912Arg)	HET	LPV	Missense	AD, AR	COL4A4	48	c.4941G>C	p.(Lys1647Asn)
c.2746+1G>T	p.?	HET	PV	Non-coding	AD, AR	COL4A3	Intron 48	c.4463-11T>C	p.?
c.1855G>A	p.(Gly619Arg)	HET	LPV	Missense	AD, AR	COL4A3	5	c.289G>A	p.(Gly97Arg)
c.3961del	p.(Asp1321Metfs*67)	HET	PV	Frame shift	AD, AR	COL4A5	3	c.160G>C	p.(Gly54Arg)

Patient Number	Sex	Age – clinical debut (y.)	Age – genetic testing (y.)	Kidney biopsy (result)	Gene	Position (Exon)
6	Male	27	48	No	COL4A3	1
10	Female	Not known	32	No	COL4A3	48
17	Female	40	53	Yes (FSGS)	COL4A4	31
29	Female	22	26	No	COL4A3	Intron 14
34	Female	42	47	Yes (AS)	COL4A3	26
36	Male	33	39	No	COL4A4	41

AD – Autosomal Dominant; AR – Autosomal Recessive; AS – Alport syndrome; COL4A3 – the alpha 3 chain of the collagen type IV molecule; COL4A4 – the alpha 3 chain of the collagen type IV molecule; COL4A5 – the alpha 5 chain of the collagen type IV molecule; Complex – Alport syndrome with complex inheritance; ESKD – end-stage kidney disease; FSGS - focal and segmental glomerulosclerosis; GFR – glomerular filtration rate; HEM – hemizygous; HET – heterozygous; HOM – homozygous; LPV – likely pathogenic variant; PV – pathogenic variant; VUS – variant of uncertain significance.

Table S2. Genetic and clinical characteristics of patients having a variant of uncertain significance

Variant			Laboratory findings at referral				Outcomes
Pathogenicity	Consequence	Inheritance	Serum creatinine (mg/dl)	eGFR (ml/min/1.73m^2)	Proteinuria (g/day)	Hematuria	ESKD (age)
VUS	In frame	AD, AR	6.46	11	4	Microscopic	Yes (28 y.)
VUS	Missense	AD, AR	1.4	48	2	Microscopic	No
VUS	Missense	AD, AR	9.46	6	NA	NA	Yes (42 y.)
VUS	Missense	AD, AR	1.02	87	NA	Absent	No
VUS	Missense	AD, AR	2.2	42	2.5	Absent	No
VUS	Non-coding	AD, AR	2.5	30	4.34	Microscopic	Yes (57 y.)

Sex	Age – genetic testing (y.)	Age – clinical debut (y.)	Kidney biopsy (result)	Gene	Position (Exon)	Nucleotide change	Amino acid change	Zygosity
Male	29	29	FSGS	COL4A3	Exon 41	c.3546_3548dup	p.(Gly1183dup)	HET
Female	41	49	No	COL4A3	Exon 44	c.3925C>T	p.(Pro1309Ser)	HET
Male	42	42	AS	COL4A4	Exon 27	c.2159C>T	p.(Pro720Leu)	HET
Male	54	54	No	COL4A4	Exon 48	c.5045G>A	p.(Arg1682Gln)	HET
Male	32	36	FSGS	COL4A4	Exon 48	c.5045G>A	p.(Arg1682Gln)	HET
Male	52	56	Angiosclerosis	COL4A4	Intron 14	c.871-3A>G	p.?	HET

Patient Number
19
26
3
23
27
35

AD – Autosomal Dominant; AR – Autosomal Recessive; AS – Alport syndrome; COL4A3 – the alpha 3 chain of the collagen type IV molecule; COL4A4 – the alpha 3 chain of the collagen type IV molecule; COL4A5 – the alpha 5 chain of the collagen type IV molecule; Complex – Alport syndrome with complex inheritance; ESKD – end-stage kidney disease; FSGS - focal and segmental glomerulosclerosis; GFR – glomerular filtration rate; HEM – hemizygous; HET – heterozygous; HOM – homozygous; LPV – likely pathogenic variant; PV – pathogenic variant; VUS – variant of uncertain significance.
